# Leptin-Mediated Changes in the Human Metabolome

**DOI:** 10.1210/clinem/dgaa251

**Published:** 2020-05-11

**Authors:** Katherine Lawler, Isabel Huang-Doran, Takuhiro Sonoyama, Tinh-Hai Collet, Julia M Keogh, Elana Henning, Stephen O’Rahilly, Leonardo Bottolo, I Sadaf Farooqi

**Affiliations:** 1 University of Cambridge Metabolic Research Laboratories and NIHR Cambridge Biomedical Research Centre, Wellcome Trust-MRC Institute of Metabolic Science, Addenbrooke’s Hospital, Cambridge, UK; 2 Service of Endocrinology, Diabetes and Metabolism, Department of Medicine, Lausanne University Hospital and University of Lausanne, Lausanne, Switzerland; 3 University Department of Medical Genetics, Addenbrooke’s Hospital, Cambridge, UK; 4 The Alan Turing Institute, London, UK; 5 MRC Biostatistics Unit, University of Cambridge, Robinson Way, Cambridge, UK

**Keywords:** leptin, metabolomics, lipids, obesity, bile acids

## Abstract

**Context:**

While severe obesity due to congenital leptin deficiency is rare, studies in patients before and after treatment with leptin can provide unique insights into the role that leptin plays in metabolic and endocrine function.

**Objective:**

The aim of this study was to characterize changes in peripheral metabolism in people with congenital leptin deficiency undergoing leptin replacement therapy, and to investigate the extent to which these changes are explained by reduced caloric intake.

**Design:**

Ultrahigh performance liquid chromatography-tandem mass spectroscopy (UPLC-MS/MS) was used to measure 661 metabolites in 6 severely obese people with congenital leptin deficiency before, and within 1 month after, treatment with recombinant leptin. Data were analyzed using unsupervised and hypothesis-driven computational approaches and compared with data from a study of acute caloric restriction in healthy volunteers.

**Results:**

Leptin replacement was associated with class-wide increased levels of fatty acids and acylcarnitines and decreased phospholipids, consistent with enhanced lipolysis and fatty acid oxidation. Primary and secondary bile acids increased after leptin treatment. Comparable changes were observed after acute caloric restriction. Branched-chain amino acids and steroid metabolites decreased after leptin, but not after acute caloric restriction. Individuals with severe obesity due to leptin deficiency and other genetic obesity syndromes shared a metabolomic signature associated with increased BMI.

**Conclusion:**

Leptin replacement was associated with changes in lipolysis and substrate utilization that were consistent with negative energy balance. However, leptin’s effects on branched-chain amino acids and steroid metabolites were independent of reduced caloric intake and require further exploration.

The administration of leptin to severely obese mice and humans lacking leptin restores body weight to normal levels, predominantly by reducing food intake (1-6). Studies in mice have shown that leptin can also modulate intermediary metabolism (7-10), which may be both centrally and peripherally mediated. Leptin reduces the expression and enzymatic activity of hepatic stearoyl-CoA desaturase-1 (SCD-1), the rate-limiting enzyme involved in the biosynthesis of monounsaturated fatty acids (FAs) (11). Moreover, leptin-deficient *ob/ob* mice with disruption of SCD-1 were found to be significantly less obese than *ob/ob* controls, suggesting that SCD-1 contributes to leptin’s effects on peripheral metabolism (11). Leptin increases sympathetic nervous system innervation of white adipose tissue in mice, enhancing lipolysis (12).

In humans, studies directly measuring leptin’s role in substrate utilization have been challenging to perform (13), given the rarity of congenital leptin deficiency and the invasive nature of adipose tissue biopsies or studies of lipid flux using stable isotopes. The potential role of leptin in regulating peripheral metabolism in humans remains unclear.

Metabolomics enables the comprehensive analysis of qualitative and quantitative changes in carbohydrate, lipid, and protein metabolites, along with their precursors and derivatives, and can be a useful tool to detect systemic changes in intermediary metabolism (14). To investigate whether leptin affects peripheral metabolism in humans, we performed metabolomic profiling in fasting serum samples from 6 children and young adults with congenital leptin deficiency before, and 1 week to 1 month after, recombinant leptin therapy. We compared metabolomic changes after leptin treatment with those seen in our previously reported study of acute caloric restriction (10% of energy requirements, mean 226 kcal/day for 48 hours) in healthy volunteers (15), identifying similarities and differences in metabolite changes after these 2 interventions. We investigated the extent to which metabolomic changes after leptin replacement therapy are consistent with reduced caloric intake and provide insights into the potential role of leptin in regulating peripheral metabolism in humans.

## Materials and Methods

### Ethical approval

This study was approved by the Cambridge Local Research Ethics Committee and conducted in accordance with the Declaration of Helsinki. Written informed consent was received from each participant (or their parent for those under 16 years). Children younger than 16 years provided oral consent.

### Experimental design

Six individuals with homozygous loss-of-function mutations in *LEP* (encoding leptin) were identified by Sanger sequencing of patients recruited to the Genetics of Obesity Study (GOOS), a cohort of over 7800 adults and children with severe, early-onset obesity, defined as body mass index (weight in kg/height in meters squared; BMI) standard deviation score (SDS) > 3 before 10 years of age, as described previously (16, 17). All 6 participants had normal renal and liver function, normal glucose tolerance (assessed after a 75 g oral glucose tolerance test) and a normal fasting lipid profile. Baseline metabolites were measured in serum samples drawn after a 12-hour overnight fast. Repeat metabolomic profiling was performed within the first month of treatment with recombinant methionyl human leptin therapy, administered as a once- or twice-daily subcutaneous injection. The initial leptin dose was calculated to achieve 10% predicted serum leptin concentration based on age, gender, and percentage of body fat (assessed by dual-energy x-ray absorptiometry, shown in Table 1) (13).

**Table 1.** Characteristics of 6 Individuals With Congenital Leptin Deficiency Before and After Leptin Treatment

Patient identifier	A	C	E	F	G	H
Age, years	18.6	3.1	13.7	8.1	2.3	7.8
Sex	Female	Male	Male	Female	Female	Male
Ethnicity	Pakistani	Pakistani	Arab	Pakistani	Turkish	Turkish
Previous leptin treatment	Yes^*a*^	No	No	No	No	No
**Baseline characteristics**						
Height, m	1.57	1.00	1.41	1.43	0.94	1.14
Weight, kg	128.7	38.8	103.0	76.2	37.2	43.8
BMI, kg/m^2^	52.2	38.8	52.2	37.4	42.1	33.7
BMI SDS (if < 18 years)	-	6.8	4.4	4.3	7.4	4.1
Fat mass, kg	70.5	21.9	57.8	59.7	19.9	23.6
Lean mass, kg	53.4	17.2	39.6	43.1	17.1	18.4
% body fat	56.7	55.4	58.7	29.1	53.7	56.1
Daily dose of leptin, mg	20	0.25	1.5	1.2	0.8	0.8
Duration of leptin treatment, days	7	28	7	28	10	7
**After leptin therapy**						
Weight, kg	126.0	38.4	101.2	74	N/A	44.2
Weight change, % baseline	-2.1	-1.0	-1.7	-2.9	N/A	0.9
BMI, kg/m^2^	51.3	37.9	51.3	36.4	N/A	34.0
Reference (if previously reported)	Refs [6, 13]	Ref [6]				

^*a*^Six-month leptin holiday prior to start of this study.

Abbreviations: BMI, body mass index; N/A, data unavailable; SDS, standard deviation score

In a follow-up study, fasting samples from children and adults within GOOS including children with homozygous loss-of-function mutations in *LEPR* (leptin receptor, n = 5), and adults with heterozygous loss-of-function mutations in *KSR2* (kinase suppressor of Ras2, n = 13) or *MC4R* (melanocortin 4 receptor, n = 27) (16, 18) were analyzed for comparison alongside samples from age- and BMI-matched individuals (11 children and 28 adults) as controls.

### Metabolomic profiling, data pre-processing, and analysis

Nontargeted metabolomic analysis of samples was performed at Metabolon, Inc. (Durham, NC) using 4 independent ultrahigh performance liquid chromatography-tandem mass spectroscopy (UPLC-MS/MS) methods as previously described (15). Details of the platform, sample processing, configuration of instruments, data acquisition, and metabolite identification have been described previously (19, 20). Data pre-processing and normalization steps for the leptin study are detailed in Supplementary Methods (21). The processed data set of 661 targeted serum metabolites were corrected for sex, age, ethnicity, and sample run day. Differential analysis of post-treatment versus pre-treatment with leptin was performed using linear modeling with empirical Bayes moderated t-statistics (LIMMA (22)) corrected for individuals, followed by multiple testing correction for metabolites (23) using the Benjamini-Hochberg method. Differential coexpression analysis was used to detect modules of interrelated metabolites whose correlation changes between pre- and post-treatment with leptin (based on DiffCoEx (24), detailed in Supplementary Methods (21)). Metabolite-set enrichment analysis was performed on preranked metabolites by LIMMA t-statistic using GSEA (25) (Supplementary Methods (21)).

For the MC4R/KSR2 study, data pre-processing, normalization, and imputation were performed by Metabolon, Inc as previously described (20). For comparison with a caloric restriction study from the same platform, metabolite fold-changes after caloric restriction compared to baseline were obtained from previously published results (15).

### Statistical analysis

Statistical tests are two-tailed unless otherwise stated, and significance of an individual test was declared at *P* < 0.05. For statistical analyses with multiple tests (differential metabolites, metabolite-set enrichment), significance was declared at a liberal false discovery rate (FDR)-adjusted *P* value < 0.2 using the Benjamini-Hochberg method. Log-scales are base 10 unless otherwise stated. Statistical analysis was performed using R statistical package.

## Results

### Leptin administration in congenital leptin deficiency leads to changes in substrate utilization

We characterized the metabolomic response to leptin replacement in severely obese people with congenital leptin deficiency. Fasting metabolome profiles were obtained before and after acute leptin treatment (duration 7 days to 1 month) in 6 children, aged from 2 to 18 years, with homozygous loss-of-function mutations in the leptin gene (*LEP*) (Table 1). Of the 6 individuals, 5 were leptin-naïve, whereas the eldest (individual A, previously reported in (6)) had previously undergone a prolonged period of leptin replacement which had been suspended 6 months prior to our study following the onset of autoantibody-mediated leptin resistance. Weight loss after acute leptin treatment was minimal, not exceeding 3% baseline weight in any individual (Table 1). The metabolome included quantification of more than 600 metabolites, divided into 7 “super-pathways” (368 lipid species, 170 amino acid derivatives, 35 nucleotide metabolites, 34 peptides, 23 cofactors and vitamins, 21 carbohydrates, and 10 tricarboxylic acid (TCA) cycle intermediates). Following pre-processing to achieve metabolite-level normalization and imputation, the data were adjusted for sex, age, ethnicity, and sample run day using a linear mixed model (detailed in Supplementary Methods (21)).

**Figure 1.**
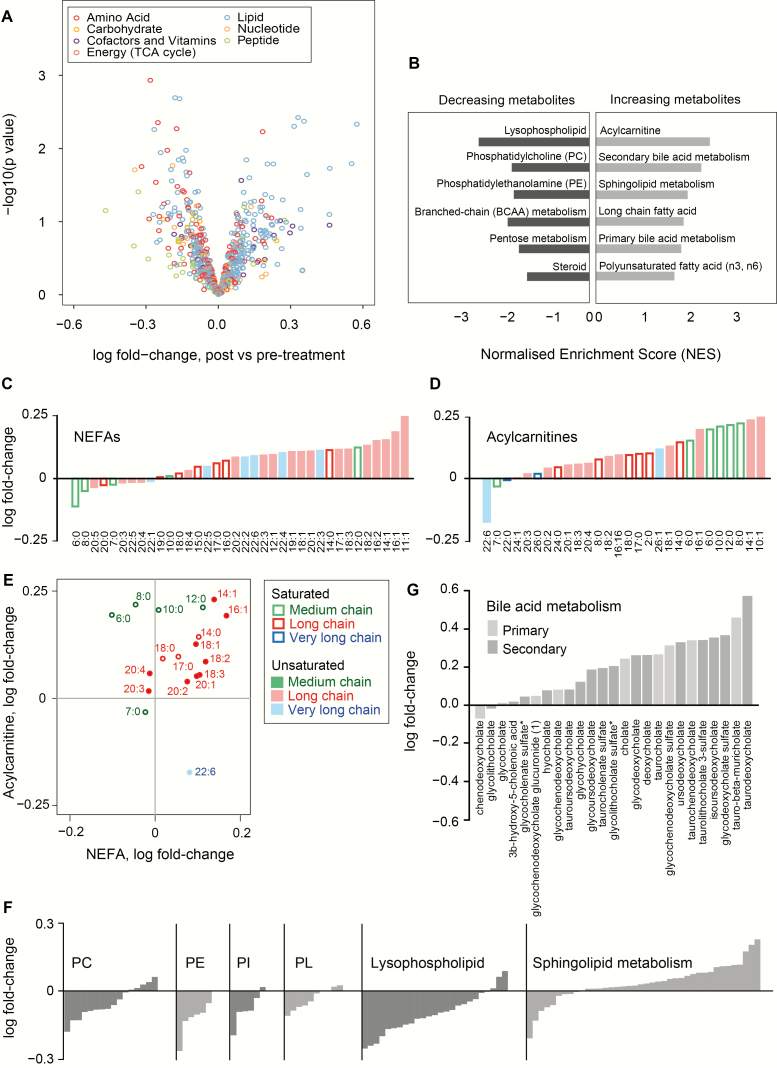
Metabolome-wide changes after acute leptin treatment in congenital leptin deficiency. **(A.)** Volcano plot showing the acute change for each metabolite upon leptin treatment (“post”) compared to before treatment (“pre-treatment”) after correcting for confounding factors. Full results are in Table S1 (21). **(B.)** Metabolite-set enrichment analysis of sub-pathway annotations showing metabolite sets with FDR *q* value < 0.2 and raw *P* value < 0.05. Full results are in Table S2 (21). **(C-E.)** Global increase in NEFAs **(C)**, acylcarnitines **(D)** and fold-change of corresponding NEFAs and acylcarnitines after leptin replacement. Filled symbols/bars indicate unsaturated, and unfilled symbols/bars represent saturated, fatty acids of different chain length: medium chain (C6-12), long chain (C13-21) and very long chain (C22 or more). **(F.)** Fold-change of metabolites after leptin treatment, illustrated for the following lipid classes: phosphatidylcholine (PC), phosphatidylethanolamine (PE), phosphatidylinositols (PI), plasmalogens (PL), lysophospholipids and sphingolipids. **(G.)** Fold-change of metabolites in the 2 sub-pathways “primary bile acid metabolism” (light grey bars) and “secondary bile acid metabolism” (dark grey bars). Metabolite-set enrichment analysis for these 2 sub-pathways is reported in Table S2, Fig. S2 (21).

We initially employed unsupervised computational approaches to investigate metabolome-wide changes upon leptin replacement. Principal component analysis of log-transformed metabolites showed an effect of inter-individual variability on the metabolomic profiles and did not consistently discriminate the pre- and post-leptin conditions (Fig. S1A). Similarly, hierarchical clustering of metabolites revealed clustering of pre- and post-treatment samples within each individual (Fig. S1B). We next used a linear model with correction for individuals to investigate the changes in each metabolite after leptin treatment (Fig. 1A; Table S1, Supplementary Methods (21)). Although individual metabolites did not reach metabolome-wide significance, we first inspected the top-ranked metabolites (nominal *P* value < 0.05, 44 metabolites; 16 up, 28 down). Of these, 14/16 increasing metabolites were in the lipids super-pathway, whereas 15/28 decreasing metabolites were lipids and 10/28 were amino acid derivatives (Table S1 (21)). In parallel, using metabolite-set enrichment analysis, we identified specific “sub-pathways” of metabolites which increased or decreased after leptin treatment (Fig. 1B, Table S2 (21)). Amongst metabolites that increased, we found an enrichment of nonesterified fatty acids (NEFAs), specifically long chain FAs and polyunsaturated fatty acids (PUFAs), acylcarnitines and sphingolipid metabolites (Figs. 1B-G). The primary and secondary bile acid metabolism sub-pathways were also enriched amongst increasing metabolites (Fig. 1G). In contrast, glycerophospholipids such as phosphatidylcholines (PCs) and phosphatidylethanolamines (PEs), as well as the lysophospholipids, were all enriched amongst metabolites that decreased (Fig. 1F), as were branched-chain amino acid (BCAA) metabolites and steroid metabolites. Collectively, these observations pointed to a shift in substrate utilization following leptin treatment, which we then tested through detailed interrogation of individual metabolite subclasses.

### Leptin administration in congenital leptin deficiency leads to divergent changes in lipid species and bile acids

Given the enrichment of distinct lipid subsets within increasing or decreasing metabolites (Fig. 1B), we systematically examined the effects of leptin replacement on a range of lipid subclasses. Although no individual lipids showed metabolome-wide statistical significance, we identified a class-wide increase in levels of FAs (medium chain FAs, long chain FAs, and PUFAs) after leptin replacement, with 27 out of 36 FAs increasing after leptin (Fig. 1C). This suggests that the transition from a leptin-deficient to a leptin-replete state promotes lipolysis, providing more substrate for FA oxidation. Monounsaturated FAs increased more than saturated and PUFAs (Kruskal-Wallis, chi-squared = 9.2, degrees of freedom = 2; *P* = 0.010; Fig. S3A (21)). There was a negative correlation between chain length and fold change of long-chain FAs with leptin (Pearson correlation = −0.59 (95% CI, −0.85, −0.08); n = 14; *P* = 0.027; Fig. S3B (21)), whereas this correlation was positive for medium-chain FAs (Pearson correlation = 0.82 (0.19, 0.97); n = 7; *P* = 0.022; Fig. S3B (21)). Due to the semi-quantitative nature of the metabolite measurements, our ability to further interrogate these changes (for example to explore the role of SCD-1 which modulates the biosynthesis of monounsaturated FAs) was limited.

In keeping with a lipolytic state, leptin replacement was also associated with a rise in circulating acylcarnitines, intermediates in FA metabolism required for their mitochondrial transport. 20/30 (67%) of acylcarnitines WERE within the leading edge of the metabolite-set enrichment analysis (Fig. 1D, Table S2 (21)). The extent of this rise correlated closely with the changes in corresponding NEFAs (long-chain, n = 12, Pearson correlation = 0.67 (95% CI, 0.16, 0.90); *P* = 0.017) (Fig. 1E). In keeping with increased beta oxidation after leptin treatment, we saw nominally significant rises in the ketone body 3-hydroxybutyrate and the corresponding 3-hydroxybutyrylcarnitine (Table S1 (21)).

Focused analysis of other lipid subclasses showed that leptin replacement was accompanied by class-wide decreases in phospholipids, including PCs, PEs, phosphatidylinositols (PI) and plasmalogens (Fig. 1F). Lysoglycerophospholipids and lysoplasmalogens, both bioactive phospholipid derivatives in which one acyl group has been removed, also reduced after leptin treatment (Fig. 1F). In contrast, leptin treatment was associated with a class-wide increase in levels of sphingomyelin (Fig. 1F; Table S2 (21)), the most abundant of the sphingolipid species, while there was no consistent effect on ceramide metabolites, synthesized by sphingomyelin hydrolysis (Table S1 (21)). Levels of sphingosine, and related metabolites dihydrosphingosine (sphinganine) and sphingosine-1-phosphate, which are key sphingolipid precursor subunits, decreased although they did not achieve nominal significance (Table S1 (21)). These observations suggest that leptin may promote the mobilization of FAs from glycerophospholipids as energy substrate, while conserving or even promoting the synthesis of sphingomyelins.

In our study, 23 out of 25 metabolites within the primary or secondary bile acid metabolism sub-pathway tended towards an increase after leptin replacement (Fig. 1G), and both of these metabolite sets were significantly enriched among increasing metabolites (Table S2, Fig. S2 (21)). The primary bile acid glycochenodeoxycholate (GCDCA) sulfate, as well as the secondary bile acids taurodeoxycholic acid (TDCA), ursodeoxycholic acid (UDCA) and its stereoisomer isoursodeoxycholic acid (IDCA) were all in the 10 highest-ranked metabolites showing the most significant changes (increase or decrease, by nominal *P* value) after leptin treatment across the metabolome, and were also found within the significantly enriched “leading edge” of the bile acid super-pathways in the metabolite-set analysis (Fig. S2 (21)). Enhanced bile acid synthesis and an increased ratio of 12α-hydroxylated bile acids (cholic acid, deoxycholic acid) to non-12α-hydroxylated bile acids (chenodeoxycholic acid, lithocholic acid, UDCA) have been associated with human insulin resistance (26), while studies have demonstrated metabolic benefit following administration of (non–12α-hydroxylated) UDCA (27). In our study, no differences were observed between primary and secondary bile acids (Fig. 1G) nor was any effect of 12α-hydroxylation apparent (Fisher’s exact test, 12α-hydroxylation status versus presence in a leading edge, odds ratio = 0.95 (0.1, 9.7), *P* = 1.0); however, UDCA and its stereoisomer were the fourth and fifth most significantly changing metabolites across the metabolome (Table S1 (21)).

### Module analysis reveals groups of metabolites coordinately regulated by leptin

Principal component analysis and hierarchical clustering analysis of metabolite profiles across the 6 individuals in our study revealed clear inter-individual differences in the metabolomic response to leptin. Despite this, there were consistent changes in metabolite classes across individuals in response to leptin treatment. To interrogate this further, we investigated groups of metabolites whose correlation profiles across the 6 individuals changed after leptin treatment. Assuming that leptin coordinates specific metabolic processes or pathways across all individuals, we would predict the presence of “metabolite modules” with a change of correlation after leptin replacement, even if the direction or magnitude of leptin’s effect varied between individuals. We performed module discovery using a differential network analysis approach based on DiffCoEx (24) which is a tool to identify modules of interrelated metabolites by detecting a change in the correlation structure between 2 groups of samples (here, after leptin treatment compared with before treatment; details are provided in Supplementary Methods (21)).

**Figure 2.**
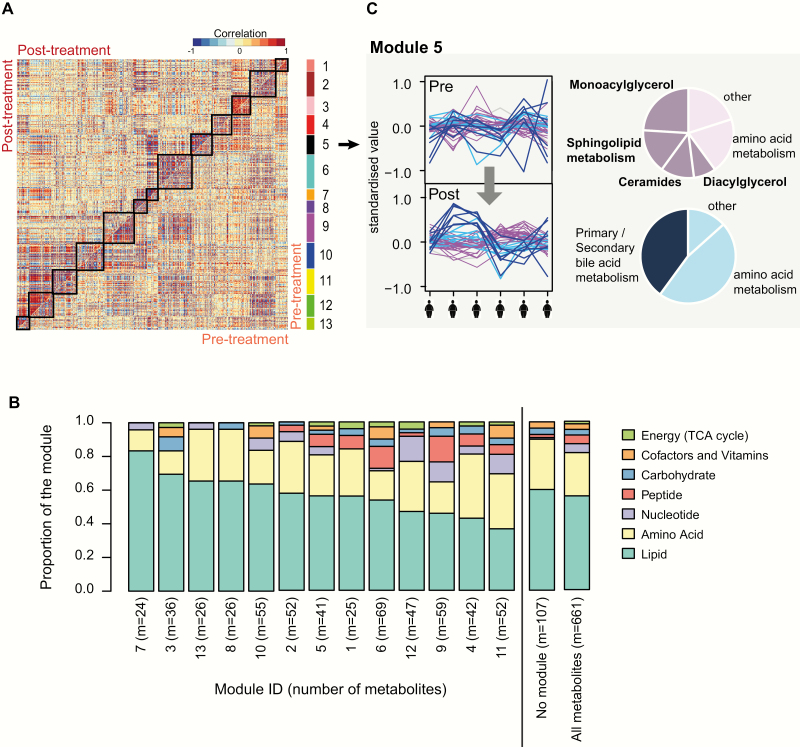
Module analysis of changes in metabolites with acute leptin replacement. **(A.)** Metabolite correlation plot indicating the 13 modules with differential correlation in post-treatment samples compared with pre-treatment samples. The upper diagonal matrix shows correlation between pairs of metabolites in the post-treatment group while the lower diagonal matrix shows the correlation between pairs of metabolites in the pre-treatment group. Modules are identified in the heat map by squares and by a color bar on the right-hand side (labeled 1 to 13). Each module consists of 1 or more submodules comprised of metabolites which are correlated or anticorrelated across the 6 individuals. For each module, the constituent metabolites and their sub-pathway annotations are provided in Table S3 (21). **(B.)** Bar plots illustrate the super-pathway composition of modules 1-13, of the remaining metabolites which were not assigned to a module, and of all the metabolites. For a more detailed description of each module, the super-pathway and sub-pathway annotation of metabolites in each module is reported in Table S4 (21), and sub-pathway enrichments among the submodules are summarized in Table S5 (21). **(C.)** Illustrative example showing module 5. The line plots display the metabolites across the 6 individuals before (“pre”) and after (“post”) leptin treatment, showing a gain of correlation after treatment. Two submodules are negatively correlated with each other (depicted in pink and blue, respectively). Pie charts show the sub-pathway composition of each submodule (details in Table S5 (21)). The composition of a second module (module 7) is illustrated in Figure S5 (21).

In total, we identified 13 metabolite modules whose degree of correlation across individuals changed after leptin treatment (Figs. 2A,B; Fig. S4, Table S3 (21)). Modules tended to show enhanced correlation after leptin treatment compared to baseline (Fig. 2A), indicating an overall loss of dysregulation among the metabolites in those modules. Analysis of the metabolite sub-pathways within each module revealed pairs of negatively correlated sub-modules with different sub-pathway compositions (Table S5 (21)). Among the top-ranked modules for sub-pathway enrichments were module 5 (Fig. 2C) and module 7 (Fig. S5, Table S5 (21)). Within module 5, leptin replacement was associated with an increase in correlation amongst a sub-module of lipid and amino acid metabolites, and this was negatively correlated with a second sub-module enriched for metabolites within the bile acid metabolism sub-pathways (Fig. 2C). This module was detected due to the gain of correlation of metabolites after leptin replacement compared with before leptin replacement, even though the direction of change differed between individuals. The enrichment of FAs and bile acid metabolites within this module implicates lipid and bile acid metabolism as coordinated, divergent downstream actions of leptin, consistent with their enrichment amongst the metabolites showing the largest individual fold changes after treatment (Figs. 1B, 1G). Similarly, the anti-correlated lipid- and steroid-enriched sub-modules within module 7 support coordinated but divergent responses of these pathways in response to leptin replacement (Table S5, Fig. S5 (21)).

### Metabolomic changes upon leptin treatment of congenital leptin deficiency overlap with those seen in acute caloric restriction

**Figure 3.**
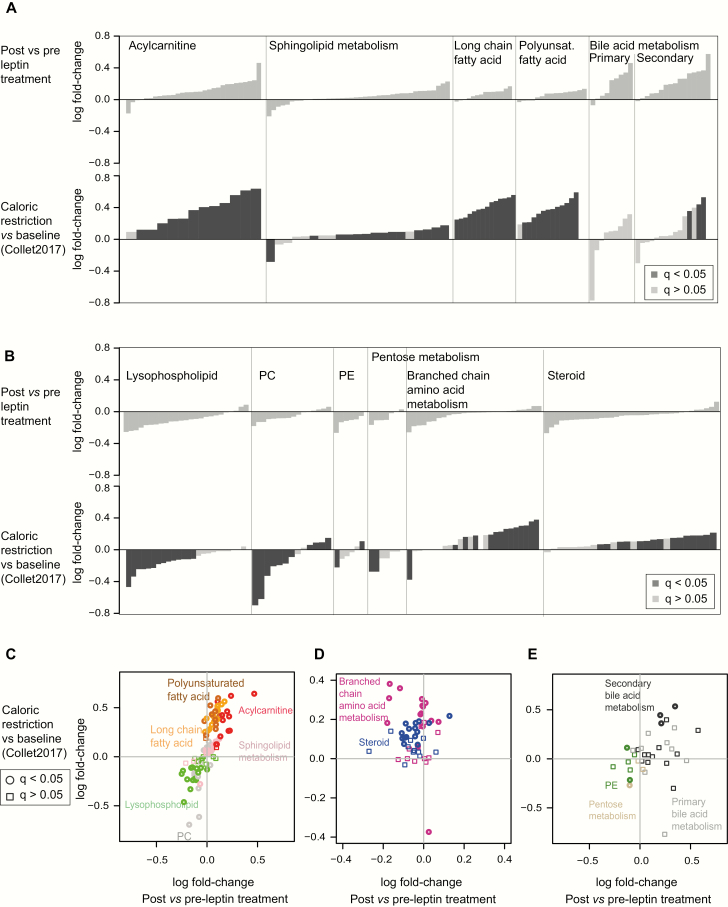
Comparison of metabolite changes associated with leptin replacement with those associated with acute caloric restriction **(A-B.)** Comparison at the level of metabolite sets based on the metabolite-set enrichment analysis of sub-pathways in Fig. 1B. In each plot, the top row illustrates fold-changes in metabolites in patients with congenital leptin deficiency post- vs pre-leptin treatment and the bottom row illustrates fold-changes upon caloric restriction versus baseline (data obtained from (15); dark columns indicate metabolites with a reported statistically significant change after caloric restriction, FDR *q* value < 0.05). Metabolites are sorted by increasing fold-change. Metabolite sub-pathways which tend towards an increase (A) or decrease (B) upon leptin treatment are shown **(also see**Fig. 1B; Table S2 (21)). **(C-E.)** Comparison at the level of individual metabolites. The scatterplots of individual metabolites show fold-change in “caloric restriction versus baseline” (as reported in (15); *y*-axis) versus acute fold-change upon leptin replacement therapy (*x*-axis). (**C**) shows the sub-pathways which have a consistent direction in the 2 studies. (**D-E**) show the sub-pathways with opposing or inconsistent directions of change between the 2 studies.

Untreated congenital leptin deficiency is characterized by hyperphagia and weight gain, representing a state of sustained positive energy balance, while acute leptin replacement rapidly suppresses food intake inducing negative energy balance. To investigate the extent of similarities between leptin replacement in congenital leptin deficiency and acute caloric restriction, we reviewed metabolomic data from our previous study of 48 hour caloric restriction in healthy, normal-weight volunteers (15), obtained using the same platform, using a similar data acquisition and target identification protocol to the present study. To enable a comparison between the 2 data sets, we identified which of the metabolites in the caloric restriction study were also quantified in the present study by matching the biochemical names and sub-pathways between the 2 annotations (details in Supplementary Methods (21)). We first examined the metabolite subgroups which, within the leptin treatment study, were enriched amongst increasing or decreasing metabolites (leptin response, FDR < 0.2 and nominal *P* value < 0.05) (Fig. 1B; Table S2 (21)) and then compared the response of the individual metabolites which had been quantified in both studies. We found that amongst metabolite subgroups that tended to increase after leptin, long chain FAs, PUFAs, acylcarnitines, glycerophospholipids, and sphingolipids all changed as a subclass in the same direction in response to acute caloric restriction, albeit with different magnitudes of response (Fig. 3A). Comparison of the behavior of individual metabolites within these subclasses confirmed that their fold-changes were highly correlated between the studies (Fig. 3C). Primary and secondary bile acids also tended to rise after acute caloric restriction, although due to high variability across individuals, most individual metabolites had not reached statistical significance in spite of large fold-changes after caloric restriction (Fig. 3D).

Amongst metabolite subclasses which decreased in response to leptin, lysophopholipids, PCs, PEs, and pentose metabolites also showed significant decreases after acute caloric restriction (Fig. 3B). In contrast, the response of steroid metabolites and BCAA metabolites was different in the 2 studies, with the majority of metabolites in both classes showing statistically significant increases in response to caloric restriction, but tending to decrease after leptin replacement (Figs. 3B, 3D). The mechanisms by which changes in energy balance affect steroid biosynthesis and metabolism are likely to be complex and variable depending on the physiological context (28).

### Individuals with severe obesity due to genetic obesity syndromes share a metabolomic signature associated with increased BMI

**Figure 4.**
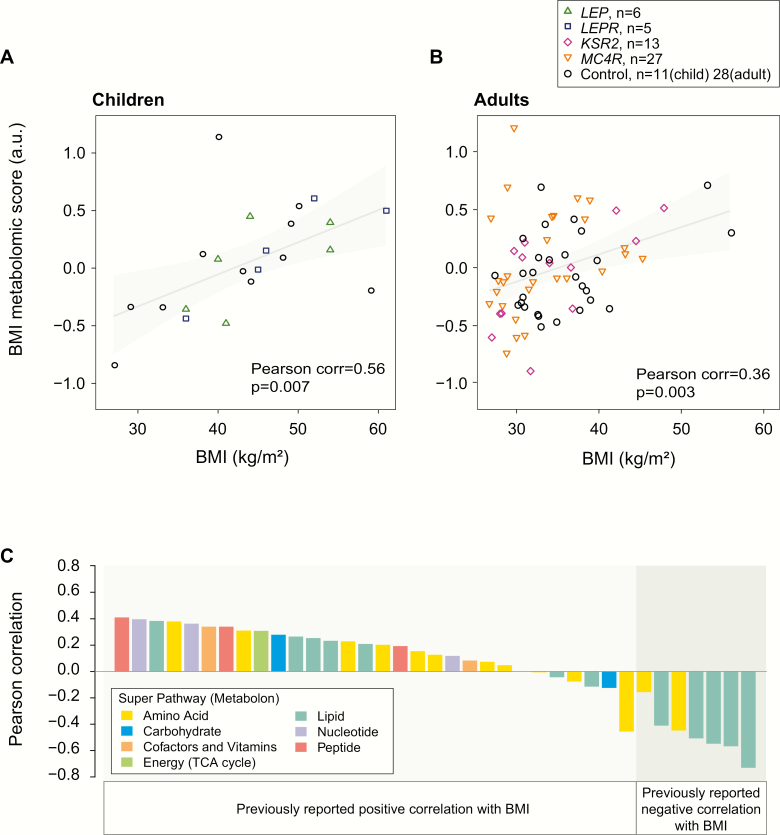
Metabolomic signature of BMI is preserved in people with genetic obesity syndromes. **(A-B.)** Scatter plots show a summary score of BMI-associated metabolites (“BMI metabolomic score”) versus BMI for (A) obese children (2–18 years of age, n = 22) and (B) adults (18-55 years of age, n = 68) with genetic obesity syndromes (harboring mutations in *LEP, LEPR, MC4R, KSR2*) and age- and BMI-matched controls. The grey line and shaded regions illustrate fitted linear regression models (95% confidence) to highlight the significant positive association with BMI. Characteristics of the study participants are summarized in Table 2. **C.** For each metabolite comprising the metabolomic BMI score, the bar plot illustrates the Pearson correlation of BMI and the metabolite value across individuals. The correlation between metabolite score and BMI is compared to correlations reported in [30].

Finally, we investigated whether changes in the metabolome that have been associated with common, polygenic obesity in other studies (29) are found in people with genetic obesity syndromes. In addition to the data from 6 people with congenital leptin deficiency, we analyzed metabolomic data from people with loss-of-function mutations in *LEPR*, *MC4R*, and *KSR2* as well as control individuals with a similar range of age, BMI, and ethnicity (Table 2, Fig. 4). We compared our data with the data from over 800 individuals obtained by Cirulli et al using a similar metabolomics platform and acquisition protocol (30), which informed the development of a model that explained 43% of the variance in BMI in their study. Here, we calculated a BMI metabolomic score based on 37 individual metabolites which overlapped with the set of 49 metabolites predicted to be associated with BMI by Cirulli et al. We found that individual metabolites within this core set of BMI-associated metabolites tended to have the same directional correlation with BMI as previously reported (30) (Fig. 4C). We calculated a “metabolomic BMI score” in each of our samples as the weighted sum of standardized metabolite values in each sample using weights (−1,1) according to the direction of correlation of these metabolites with BMI in Cirulli et al. Our BMI metabolomic score correlated significantly with BMI within the obese to severely obese range in children (Fig. 4A; n = 22, Pearson correlation = 0.56 [95% CI, 0.18, 0.79]; *P* = 0.007) and in adults (Fig. 4B; n = 68, Pearson correlation = 0.36 [0.13, 0.55]; *P* = 0.003). We observed similar correlations between BMI and BMI metabolomic score within the different genetic disorders studied (Supplementary Methods (21)); there was no consistent change in the metabolomic BMI score after leptin treatment (*P* = 0.69; paired Wilcoxon signed rank). Our findings in severe obesity due to genetic obesity syndromes support the derivation and use of a metabolomic signature of the obese state within the obese to severely obese range of BMI, both among children and adults.

**Table 2.** Characteristics of Participants With Genetic Obesity Syndromes

Genetic obesity syndrome	*LEP*	*LEPR*	Control	*KSR2*	*MC4R*	Control
Number of participants	6	6	12	13	31	28
Age group (child/adult)	6^*a*^/0	5^*a*^/1	11^*a*^/1	0/13^*b*^	4/27^*b*^	0/28^*b*^
Gender (M/F)	3/3	3/3	6/6	5/8	13/18	11/17
Age, years	8.50 (2.49)	14.2 (1.96)	12.6 (2.51)	32.0 (3.71)	32.7 (2.00)	39.8 (1.73)
Body mass index, kg/m^2^	42.8 (3.08)	45.2 (3.48)	39.8 (2.67)	34.5 (1.87)	35.1 (1.33)	35.6 (1.20)
Ethnicity						
*Caucasian*	*0*	*3*	*5*	*13*	*19*	*20*
*Pakistani*	*3*	*1*	*6*	*0*	*11*	*0*
*Turkish*	*2*	*2*	*1*	*0*	*0*	*0*
*Arab*	*1*	*0*	*0*	*0*	*0*	*0*
*Afro-Caribbean*	*0*	*0*	*0*	*0*	*0*	*1*
*Mixed*	*0*	*0*	*0*	*0*	*1*	*0*
*Unknown*	*0*	*0*	*0*	*0*	*0*	*7*

Data are presented as mean (SEM)

^*a*^18 years and younger, as shown in Fig. 4A “Children.”

^*b*^18 years and older, as shown in Fig. 4B “Adults.”

## Discussion

In this study, we used metabolomic profiling to study people with congenital leptin deficiency, before and after leptin treatment. Principal component analysis and hierarchical clustering analysis of metabolite profiles across the 6 individuals revealed pronounced inter-individual variation in the metabolomic response to leptin, which may be partly attributable to differences in age, duration of therapy, and previous leptin exposure. The small study size (n = 6) reflects the rarity of this condition; there may be further differences that could not be detected in this study. However, despite this variation, and despite the small study size, we found consistent changes in metabolite classes across individuals in response to leptin. Leptin replacement resulted in a class-wide increase in NEFAs, an increase in cognate acylcarnitines, and a significant increase in beta-hydroxybutyrate, providing evidence that leptin promotes lipolysis and FA oxidation in humans. Decreases in multiple glycerophospholipid classes, including PCs, PIs, PE, and plasmalogens, suggests that not only triglycerides but also glycerophospholipids are broken down in response to leptin administration. Metabolomic profiling after leptin treatment therefore demonstrates that in humans, as in mice, leptin elicits a shift towards lipid catabolism. These observations are consistent with the observations that weight loss after leptin treatment in children with congenital leptin deficiency is predominantly due to loss of fat mass (98%) (13), contrasting with loss of both fat mass (75%) and fat-free mass observed with weight loss due to caloric restriction in common obesity (31).

In keeping with leptin’s anorectic effects (32), the metabolomic response to acute leptin replacement in congenital leptin deficiency showed many similarities to that observed after 48 hours of caloric restriction in healthy volunteers, with both interventions driving increased levels of acylcarnitines, increased FAs, and decreased lysophospholipids (30, 33). Similar changes have been observed after weight loss (34), in keeping with leptin’s role in the weight-reduced state (35). Collectively, these data suggest that many of the effects of leptin on human lipid metabolism are attributable to reduced food intake and negative energy balance. Our findings in humans align with experiments in leptin-deficient *ob/ob* mice pair-fed to leptin-treated *ob/ob* mice, which showed that leptin’s effects on peripheral metabolism are predominantly explained by changes in food intake (36).

Leptin replacement and acute caloric restriction had divergent effects on the BCAA-related and steroid-related metabolite clusters. Elevated levels of BCAAs, observed here after caloric restriction but not leptin administration in congenital leptin deficiency, have been repeatedly associated with insulin resistance, diabetes, and cardiovascular disease in multiple cohorts (37, 38). The mechanisms underpinning these associations are incompletely understood; according to one model, generation of short-chain acylcarnitines via enhanced BCAA catabolism may lead to “clogging” and reduced efficiency of the beta oxidation machinery. Failure of leptin to activate BCAA catabolism supports a model in which leptin specifically activates peripheral lipid metabolism with minimal or no effect on protein catabolism, in contrast to caloric restriction where both substrates are affected. Similarly, the divergent effects of leptin replacement and acute caloric restriction on steroid metabolites may reflect an effect of leptin on steroidogenic pathways. This observation is complicated, however, by the effects of both glucocorticoids and sex steroids on peripheral metabolism, substrate utilization, and body composition, particularly given the different age, sex, pubertal status, and BMI of the participants in the 2 studies.

While absolute levels of many metabolites, including bile acids, are difficult to interpret, both primary and secondary bile acid metabolites were significantly enriched amongst metabolites that increased after leptin replacement. In *ob/ob* and *db/db* mice and the Zucker *fatty* rat, impaired hepatic cholesterol catabolism, decreased bile acid synthesis and transport and impaired biliary clearance have all been reported; additionally, expression of key genes involved in bile acid synthesis, including *Cyp7a1*, is reduced in *ob/ob* mice compared with wild-type controls (39-41). Leptin replacement in *ob/ob* mice contributes to intestinal cholesterol absorption and increased levels of bile acids (42). In this study, a comparable increase in bile acid levels in humans after leptin replacement may similarly reflect a drive towards cholesterol catabolism.

Finally, we demonstrated that a BMI metabolomic score, initially derived from a large cohort studied longitudinally (30), is robust even at the extreme upper end of the BMI spectrum, including individuals with defined genetic obesity syndromes and severely obese controls, both in adults and children. Our findings therefore validate this score as a robust signature of BMI well into the pathological range. Metabolites associated with increasing BMI include branched-chain and aromatic amino acids and metabolites involved in nucleotide metabolism, including urate and pseudouridine (43). Understanding the mechanisms by which these metabolites directly or indirectly influence fundamental processes involved in substrate utilization may provide new insights and potential therapeutic targets for obesity-associated metabolic disease.

## Abbreviations

BCAA: branched-chain amino acid
BMI: body mass index
BMI-SDS: BMI standard deviation scores
FA: fatty acid
FDR: false discovery rate
GOOS: Genetics of Obesity Study
NEFA: nonesterified fatty acid
PC: phosphatidylcholine
PE: phosphatidylethanolamine
PI: phosphatidylinositol
PUFA: polyunsaturated fatty acid
SCD-1: stearoyl-CoA desaturase-1
TCA: tricarboxylic acid
UDCA: ursodeoxycholic acid
UPLC-MS/MS: Ultrahigh performance liquid chromatography-tandem mass spectroscopy

## Notes

***Financial Support:*** This study was supported by funding from the Wellcome Trust (207462/Z/17/Z), the NIHR Cambridge Biomedical Research Centre and the Bernard Wolfe Health Neuroscience Endowment (to I.S.F.), the Swiss National Science Foundation (P3SMP3-155318, PZ00P3-167826, to T.H.C.), the Uehara Memorial Foundation (to T.S.), the Alan Turing Institute under the Engineering and Physical Sciences Research Council grant EP/N510129/1 (to L.B.) and an NIHR Clinical Lectureship (to I.H-D.). The authors thank Paul Kirk and Angelos Alexopoulos for suggestions and insightful comments regarding the KNN-TN software during pre-processing and imputation. Clinical studies were performed on the IMS Translational Research Facility, which is funded by Wellcome (208363/Z/17/Z). This work was supported in part by the NIHR Rare Diseases Translational Research Collaboration. The views expressed are those of the authors and not necessarily those of the NHS or the NIHR.

***Author Contributions:*** I.S.F. and S.O’R. conceived the study; J.M.K., E.H., T-H.C. and I.S.F. conducted the clinical studies; K.L., I.H-D. and L.B. designed and performed statistical analyses and analyzed data; K.L., I.H-D., T-H.C., T.S., L.B. and I.S.F. contributed to study design and interpretation of results. K.L., I.H-D. and I.S.F. wrote the paper. All authors contributed to and approved the final version of the paper.

## Additional Information

***Disclosure Summary*:** The authors have no conflicts of interest relevant to this article to disclose.

***Data Availability:*** All tables of analyzed results are available as Supplementary Tables and Supplementary Figures (21). The metabolomic datasets generated during the current study are available from the corresponding author on request.
